# Development of the Intestinal RNA Virus Community of Healthy Broiler Chickens

**DOI:** 10.1371/journal.pone.0150094

**Published:** 2016-02-25

**Authors:** Jigna D. Shah, Prerak T. Desai, Ying Zhang, Sarah K. Scharber, Joshua Baller, Zheng S. Xing, Carol J. Cardona

**Affiliations:** 1 Veterinary and Biomedical Sciences, University of Minnesota, Saint Paul, Minnesota, United States of America; 2 Minnesota Supercomputing Institute, University of Minnesota, Minneapolis, Minnesota, United States of America; 3 Microbiology and Molecular Genetics, University of California—Irvine, Irvine, California, United States of America; Wageningen University and Research Centre, NETHERLANDS

## Abstract

Several RNA viruses such as astrovirus, rotavirus, reovirus and parvovirus have been detected in both healthy and diseased commercial poultry flocks. The aim of this study was to characterize (a) the development of the RNA viral community in the small intestines of healthy broiler chickens from hatch through 6 weeks of age (market age) and (b) the contribution of the breeder source vs. bird age in development of the community structure. Intestinal tissue samples were harvested from breeders and their progeny, processed for viral RNA extraction and sequenced using Illumina Hiseq sequencing technology resulting in 100 bp PE reads. The results from this study indicated that the breeder source influenced the RNA viral community only at hatch but later environment i.e. bird age had the more significant effect. The most abundant RNA viral family detected at 2, 4 and 6 weeks of age was Astroviridae, which decreased in abundance with age while the abundance of Picornaviridae increased with age.

## Introduction

Vertebrates are host to large communities of bacteria, fungi, archaea and viruses collectively known at the microbiome. Microbial communities, especially the bacterial constituent, are known to contribute to the healthy states of their hosts. In contrast, eukaryotic viruses that infect the host and inflict cellular damage, have commonly been studied as pathogens rather than as members of a healthy microbiota. But, as evidence emerges that there are diverse viral communities in healthy vertebrate hosts, that paradigm is shifting [1]. With new methods to study the virome [2], the picture that is beginning to emerge is that of eukaryotic viruses as part of a healthy microbiota and part of a host’s genetic identity [3].

Although viruses are an essential component of the normal microbiota of the gut, there are also virulent pathogens that can cause enteric disease. Most of these viruses have RNA genomes which infect the host and cause lesions. Rotaviral diarrheal diseases, for example, are a major cause of illness among children, calves, foals, pigs, chicks and poults [4,5]. In addition to rotaviruses, other RNA viral families have been linked to enteritis including Astroviridae, Reoviridae, Coronaviridae and Parvoviridae [6–9]. Despite links to disease, these same viral families have been detected in the gastrointestinal tracts of healthy animals of the same species [10–13] suggesting that the relationship between host and virus likely includes both symbiosis and disease, and some balance between the two states.

Although eukaryotic viruses may not be in an entirely symbiotic relationship with their hosts, it is evident that not all infections are symptomatic. To investigate the dynamics of the members of the RNA virome, we examined the RNA viruses in the gastrointestinal tract of healthy chickens. As a host, chickens can be infected with several enteric virus families and are susceptible to clinical enteritis. In our previous study, we developed a method to process the whole intestine, both tissue and contents, using high-throughput next generation sequencing to characterize the RNA viral community [2]. In this study, using the established method, we followed broiler chickens from hatch to market as well as their dams to understand how RNA viral communities develop and change as the host ages and how they relate to enteric health.

## Materials and Methods

### Study design

Tissues were collected post mortem from poultry raised on commercial farms (source: GNP company, St. Cloud, MN). At each sample collection timepoint except for the hatch timepoint, 30 birds were randomly selected, necropsied and examined for grossly evident lesions. The small intestines of six broiler breeder hens from a single flock were collected post mortem and numbered A1—A6. Small intestines from the progeny of the same breeder flock were collected at hatch (H1—H6). The remaining cohort of chicks from the sampled breeder flock was placed in the lower story of a two-story commercial broiler house (hereafter called the monitored flock). A second flock was placed at the same time on the second story (hereafter called the control flock). The control flock was composed of chicks from five breeder sources. The monitored and control flocks received water from the same source and were given the same feed according to standard practice. Intestines were collected from six birds post mortem from the monitored and control flocks each when the birds were 2 weeks (monitored: 2W_1–2W_6 and control: 2W_C1–2W_C6), 4 weeks (monitored: 4W_1–4W_6 and control: 4W_C1–4W_C6) and 6 weeks (monitored: 6W_1–6W_6 and control: 6W_C1–6W_C6) of age.

### Sample collection, processing and RNA extraction

The jejunum and ileum along with their contents were harvested from each carcass and processed as described previously [2]. RNA extraction from each viral pellet was performed as described previously [2] with one modification. The viral pellet was treated with 2 units of DNAaseI (New England Bio Labs, Ipswich, MA) per 100 μl of reaction and incubated at 37°C for 10 min, before RNA extraction. The quality of the total RNA was measured with a Nanodrop 2000 (ThermoScientific, Wilmington, DE).

### Sequencing

Total RNA from each sample was converted to Illumina sequencing libraries using Illumina’s TruSeq RNA Sample Prep Kit v2 according to manufacturer’s instructions. Final library size distribution was validated and indexed, and the libraries from paired ends were pooled and size-selected at 320 bp +/- 5% using Caliper’s XT instrument giving an average insert size of approximately 200 bp. The pooled libraries were sequenced on a Hiseq 2000 with 100 bp PE run. De-multiplexed FASTQ files were used for subsequent analysis. The data have been deposited with links to BioProject accession number PRJNA310100 in the NCBI BioProject database (https://www.ncbi.nlm.nih.gov/bioproject/).

### BLAST and taxonomy mapping

**Fig 1:**
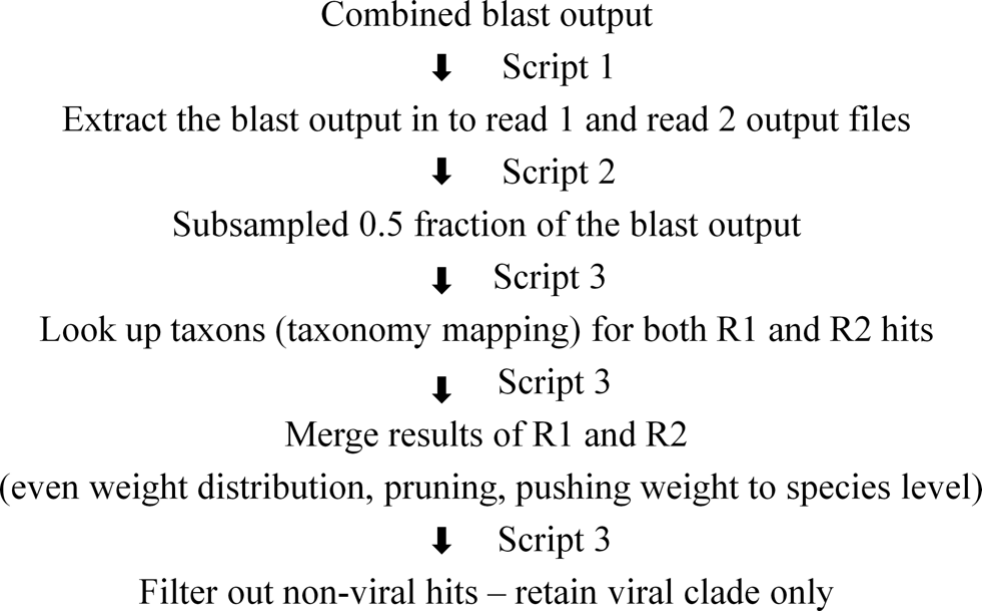
Brief schematic representation of the steps involved in taxonomy mapping after the blastn. Scripts 1, 2 and 3 were custom developed python scripts.

The quality of sequences from each of the samples was evaluated using FastQC. The paired reads from each sample were concatenated and one unique copy of each fragment was retained. The joined fragments were split to regenerate the forward (split R1) and reverse (split R2) reads. AbokiaBLAST, a commercial parallel implementation of NCBI BLAST was used for all blast searches against the NT database downloaded from NCBI in August 2013. The paired reads were blasted independently against the NT database using the program’s default parameters. Due to computational resource limitations and the large dataset size, blast could not be completed on the samples A3, H3, H4, 4W_C2 and 6W_C2 and hence these samples were excluded from further analysis. There were numerous indications of sample saturation allowing the blast output to be subsampled at the 50% level to improve analysis speed. The assignment of taxonomic ID associated with each sequence and reconciliation of information from paired ends was performed using custom python scripts as described previously [2]. The brief schematics of the steps involved are shown in Fig 1. The reconciled output was used to distribute weight evenly to leaf nodes and the leaf nodes with weights below the cutoff were pruned [2]. MEGAN 4 software was used to visualize taxonomic weights and extract the information at family, genus and species levels.

### Descriptive statistics

Rarefaction curves, alpha diversity measures, beta diversity measures and analysis of molecular variance (AMOVA) at the genus level were computed using mothur v.1.33.3 [14].

Each sample had a unique sequencing depth and library size resulting in different numbers of reads with hits to RNA viruses for each sample. Table 1 lists the number of total and unique reads, and the reads with hits to viral clades and RNA viral families for half of the blast output. The H samples had the lowest reads with hits to RNA viruses. Thus, the rarefaction analysis for the H samples was done at a frequency of 1,000 sequences while for all other samples it was done at a frequency of 10,000 sequences.

**Table 1:** Number of reads after each step of processing and taxonomy mapping for each sample.

			Reads with hits to	
Sample	Total reads	Unique reads	Viral clades	RNA viruses
**A1**	11017425	6088241 (55.26%)	885875 (14.55%)	650184 (73.39%)
**A2**	16265511	4635792 (28.50%)	816548 (17.61%)	384463 (47.08%)
**A4**	16399570	6085651 (37.11%)	974485 (16.01%)	950742 (97.56%)
**A5**	17223661	4269257 (24.79%)	974785 (22.83%)	931158 (95.52%)
**A6**	15225450	4311111 (28.32%)	768149 (17.82%)	214075 (27.87%)
**H1**	10464690	2898615 (27.70%)	754423 (26.03%)	137668 (18.25%)
**H2**	10245546	2862520 (27.94%)	776793 (27.14%)	239361 (30.81%)
**H5**	10895021	3840389 (35.25%)	770677 (20.07%)	236349 (30.67%)
**H6**	13420086	4541611 (33.84%)	775962 (17.09%)	244508 (31.51%)
**2W_1**	10333772	5165536 (49.99%)	944721 (18.29%)	844186 (89.36%)
**2W_2**	9839085	3082541 (31.33%)	923404 (29.96%)	796148 (86.22%)
**2W_3**	10389368	3869490 (37.24%)	977582 (25.26%)	958123 (98.01%)
**2W_4**	9575047	3431132 (35.83%)	967227 (28.19%)	947589 (97.97%)
**2W_5**	14688703	4230735 (28.80%)	905403 (21.40%)	867690 (95.83%)
**2W_6**	12672950	4157775 (32.81%)	984353 (23.67%)	976642 (99.22%)
**4W-1**	14350619	3102900 (21.62%)	840061 (27.07%)	462281 (55.03%)
**4W-2**	10009273	3310023 (33.07%)	800462 (24.18%)	405011 (50.60%)
**4W-3**	11145873	2608773 (23.41%)	876472 (33.60%)	651308 (74.31%)
**4W-4**	9877312	2232649 (22.60%)	688244 (30.83%)	335337 (48.72%)
**4W-5**	9809959	3170622 (32.32%)	876612 (27.65%)	774810 (88.39%)
**4W-6**	10725614	2416105 (22.53%)	791286 (32.75%)	565373 (71.45%)
**6W_1**	18672470	4564955 (24.45%)	768565 (16.84%)	531352 (69.14%)
**6W_2**	12005002	5856543 (48.78%)	671770 (11.47%)	445514 (66.32%)
**6W_3**	14941416	3475566 (23.26%)	678542 (19.52%)	351740 (51.84%)
**6W_4**	13123757	4411398 (33.61%)	678641 (15.38%)	500855 (73.80%)
**6W_5**	11614451	2645043 (22.77%)	796965 (30.13%)	595790 (74.76%)
**6W_6**	15843770	3555248 (22.44%)	696556 (19.59%)	516302 (74.12%)
**2W-C1**	10732185	2794918 (26.04%)	974146 (34.85%)	943289 (96.83%)
**2W-C2**	11324836	2929375 (25.87%)	986488 (33.68%)	973515 (98.68%)
**2W-C3**	13476810	3255780 (24.16%)	944175 (29.00%)	867467 (91.88%)
**2W-C4**	13192878	3915756 (29.68%)	946192 (24.16%)	913804 (96.58%)
**2W-C5**	9841926	2962819 (30.10%)	932038 (31.46%)	898781 (96.43%)
**2W-C6**	12595010	3539277 (28.10%)	941149 (26.59%)	907729 (96.45%)
**4W-C1**	8177348	5626179 (68.80%)	905884 (16.10%)	776345 (85.70%)
**4W-C3**	11482647	4212528 (36.69%)	855312 (20.30%)	789819 (92.34%)
**4W-C4**	14758087	4072377 (27.59%)	959787 (23.57%)	903149 (94.10%)
**4W-C5**	10606837	3005496 (28.34%)	983173 (32.71%)	968998 (98.56%)
**4W-C6**	8466996	2983033 (35.23%)	798615 (26.77%)	719964 (90.15%)
**6W_C1**	17461426	4414948 (25.28%)	746407 (16.91%)	697474 (93.44%)
**6W_C3**	15585444	3548674 (22.77%)	744169 (20.97%)	270349 (36.33%)
**6W_C4**	8252549	5970853 (72.35%)	669081 (11.21%)	485234 (72.52%)
**6W_C5**	10663209	2913787 (27.33%)	859389 (29.49%)	811411 (94.42%)
**6W_C6**	16242932	3470079 (21.36%)	814756 (23.48%)	410725 (50.41%)

The first two data columns represent the number of total reads and unique reads, and the last two data columns represent the number of reads with hits to viral clade and RNA viral families for only half fraction of the blast output. The percent value in each column represents the percent reads from the previous column.

**Fig 2:**
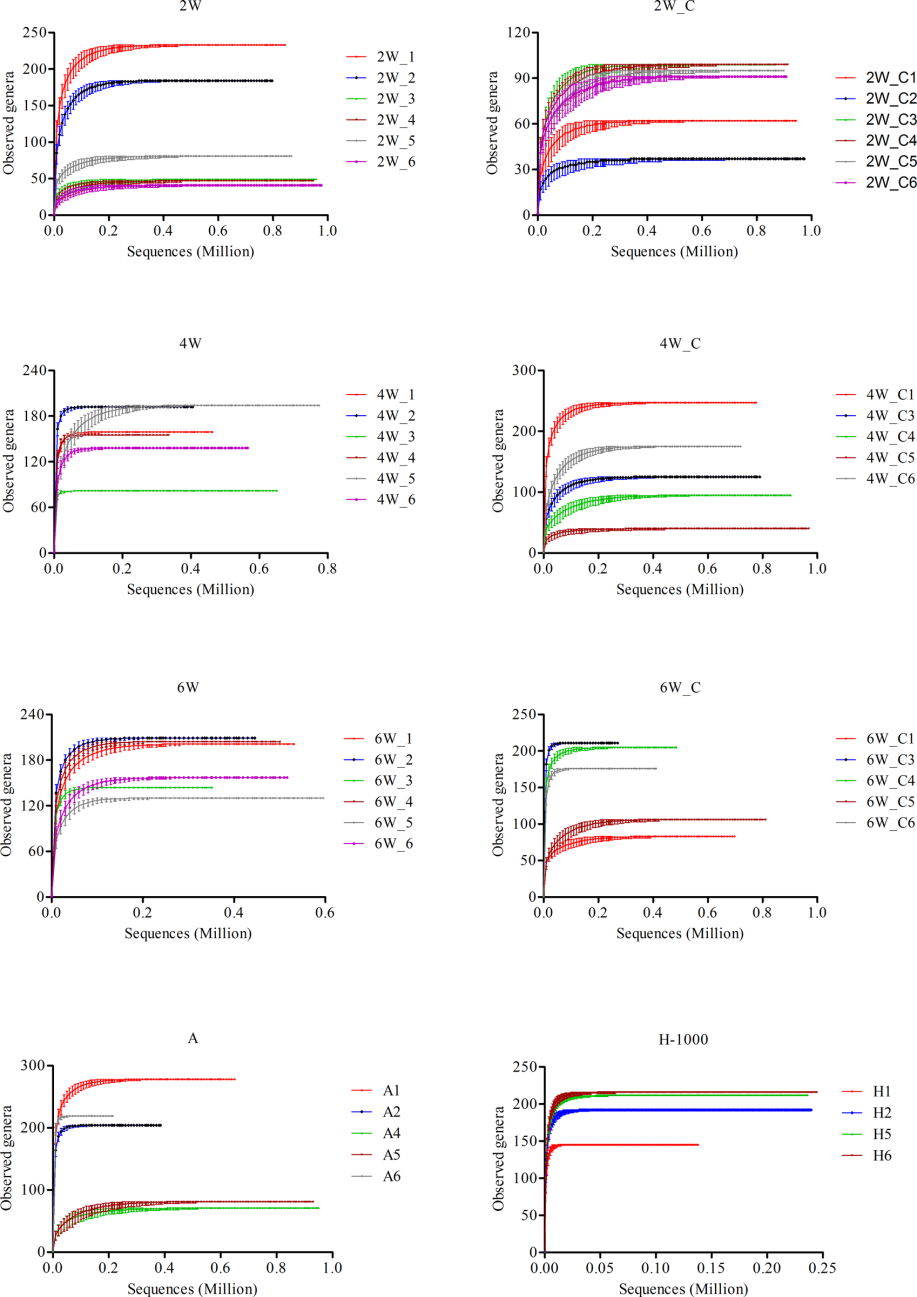
Rarefaction curves for all samples. The curves for each sample were generated by varying subsampling level with intervals of 10,000 sequences except for hatch (H) samples which used intervals of 1,000 sequences. The Y-axis represents the number of genera and the X-axis represents the number of sequences.

To compare the alpha diversity across samples, the number of reads with hits to RNA viruses for each sample was standardized to the number of reads in the smallest library i.e. sample H1 (Table 1). The alpha diversity measures, Chao I index, Sobs (number of observed genera) and the invsimpson index were computed (average of 1000 replicates) on 137,668 randomly sampled sequences from each sample. However, in all samples (except H), this level of sub-sampling failed to saturate the number of genera present in the datasets likely leading to an underestimation of alpha diversity (Fig 2). To compare the alpha diversity of the remaining samples, the number of reads with hits to RNA viruses was sub-sampled to the number of reads in the 6W_C3 library i.e. 270,349 (Table 1). Sub-sampling at 137,668 sequences will be called small sub-sampling and sub-sampling at 270,349 sequences will be called large sub-sampling. The Chao I, Sobs and the invsimpson indices for the non-H samples were computed on the large sub-sample. Two-way ANOVA on the invsimpson indices was done using the commercially available GraphPad Prism 5 software to evaluate the respective impacts of bird age vs. breeder source (statistical model of ‘bird age + breeder source + breeder source*age’) on progeny viral diversity.

To measure beta diversity, the Bray-Curtis dissimilarity index [15] was computed on the small and large sub-samples from each sample. These dissimilarity matrices were used as input for ordination analyses and non-metric multidimensional scaling (NMDS) plots were created. To further determine if the spatial separation between the groups observed was statistically significant, analysis of molecular variance (AMOVA) was performed and *p* < 0.05 considered significant.

### Inferential statistics

Differential abundance analysis at the family level was computed using edgeR package in R v.3.1.2 [16] with the relative log expression (RLE) method to normalize the data as described previously [17]. The statistical model of ‘bird age + breeder source’ was used. The RLE normalized data was also used for abundance profiles. The balanced design considered for the two-way ANOVA and differential abundance analysis with edgeR involved test birds from breeder A at 2 (2W), 4 (4W) and 6 (6W) weeks of age, and control birds from breeder A at 2 (2W_C), 4 (4W_C) and 6 (6W_C) weeks of age.

## Results

### Flock health

The breeder, monitored and control flocks were within normal production parameters for the source company. There were no gross lesions observed in the 30 bird samples collected from the breeder hens, nor from the birds in the monitored and control flocks at 2, 4 or 6 weeks of age.

### Diversity of RNA viruses in individual birds

**Fig 3:**
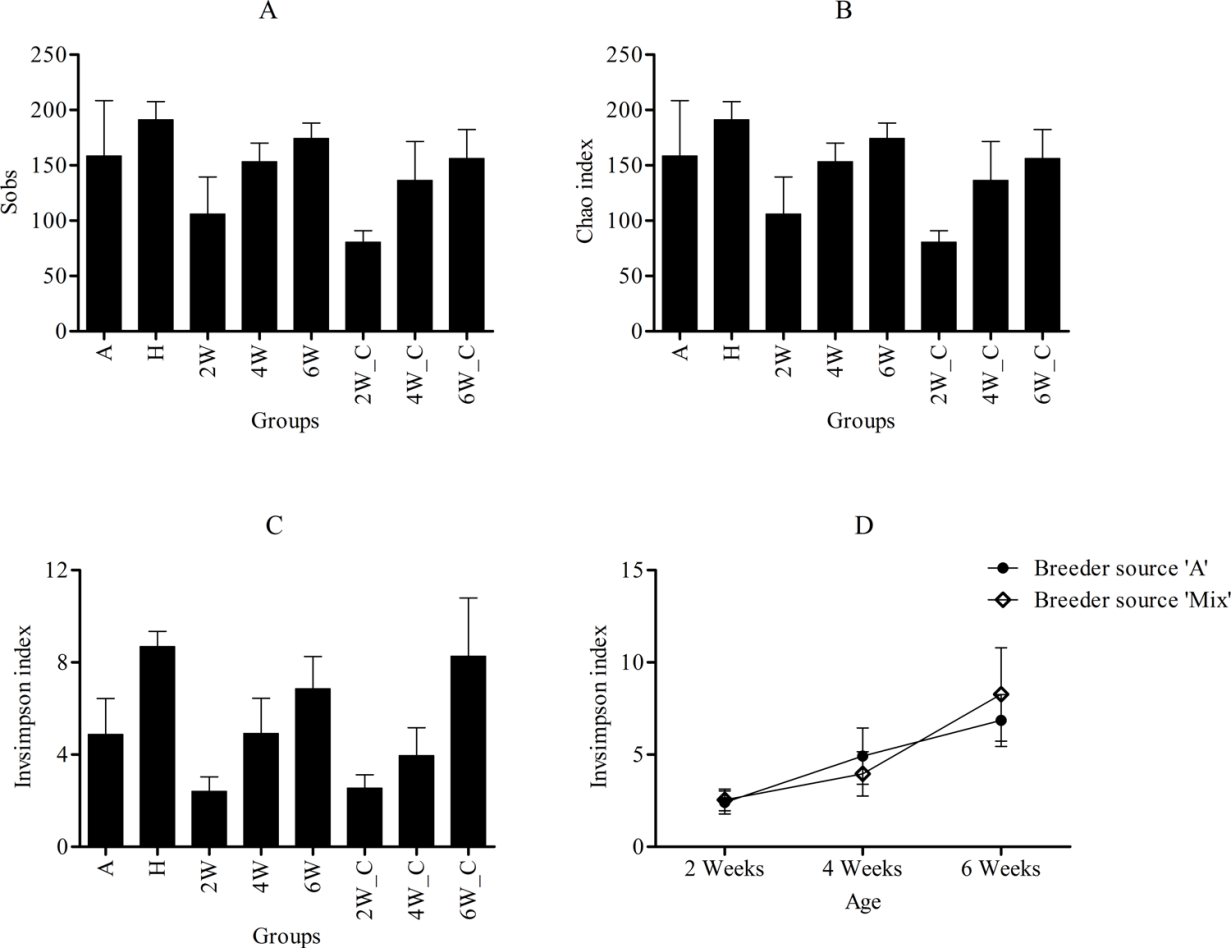
Representation of alpha diversity indices. (A) Number of observed genera (Sobs) for each group (B) Estimated richness (Chao) for each group (C) Diversity (Invsimpson) for each group (D) Two-way ANOVA on the Invsimpson indices where the Y-axis represents the Invsimpson index and the X-axis represents the age group. These alpha diversity indices were calculated on 137,668 randomly sampled sequences from H group and 270,349 randomly sampled sequences from the remaining groups.

For all the samples, increased sampling caused the rarefaction curves to plateau indicating that the majority of RNA viral diversity had been measured (Fig 2). The Chao I index, the richness estimator, was similar to Sobs, the number of observed genera (Fig 3A and 3B). The number of genera detected as well as the diversity increased with age in birds from both test and control flocks (Fig 3C). Further, a two-way analysis of variance performed on the invsimpson indices confirmed that age had a significant effect with a *p*-value of 0.004 whereas breeder source and the interaction (breeder source*age) had no significant effect indicating that the major driver of viral diversity was age (Fig 3D).

### Comparison of composition and structure of RNA viral communities of all birds

**Fig 4:**
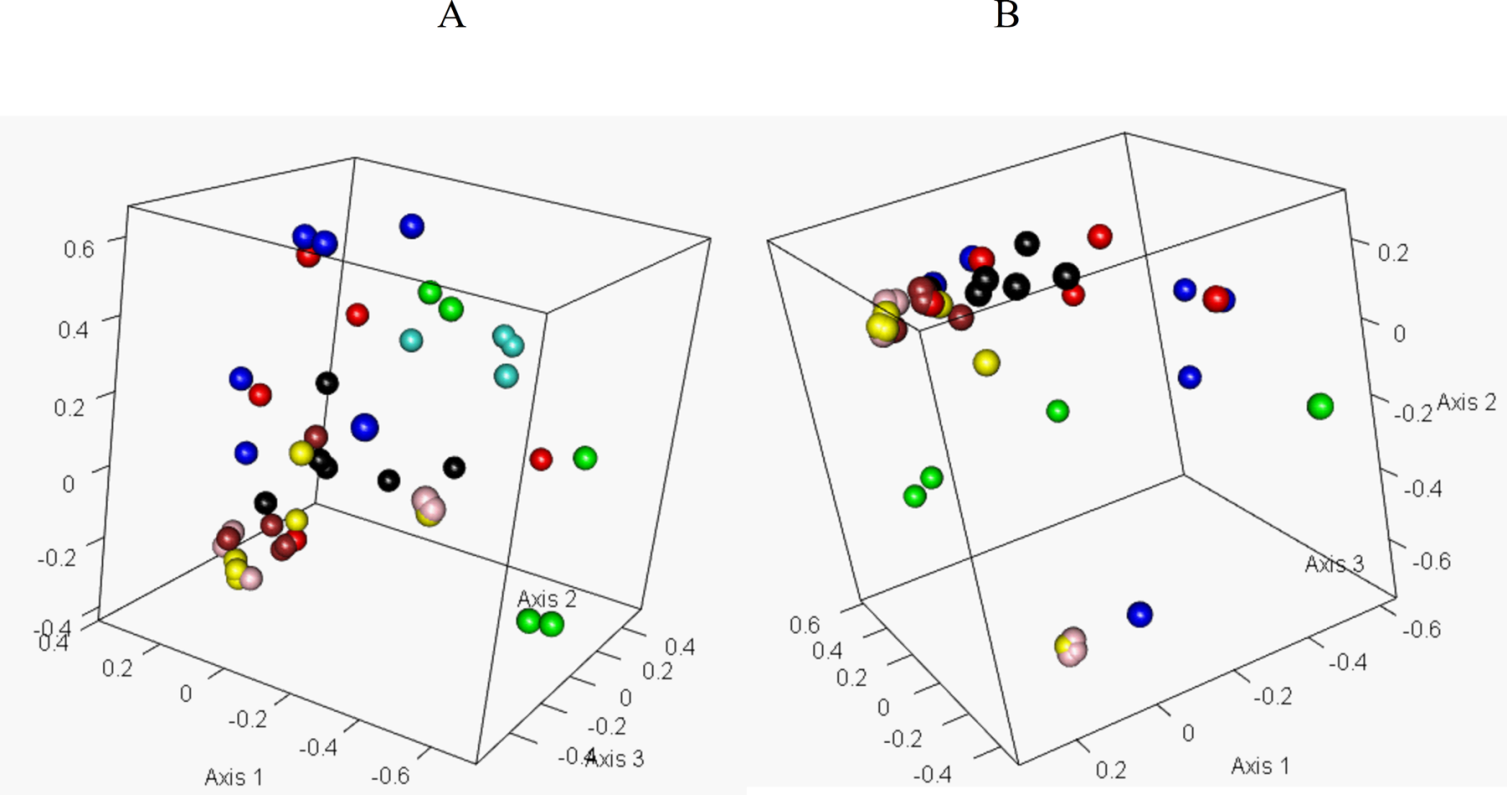
Ordination analysis. Non-metric multidimensional scaling plots to visualize the community distances calculated using Bray-Curtis dissimilarity index on (A) 137,668 sequences sub-sampled from each sample and (B) 270,349 sequences sub-sampled from each sample. The color legends are A: Green, H: Turquoise, 2W: Yellow, 2W_C: Pink, 4W: Black, 4W_C: Brown, 6W: Blue, and 6W_C: Red.

The Bray-Curtis dissimilarity index matrices were used as input for ordination analyses. Non-metric multidimensional scaling (NMDS) plots were created resulting in R-squared values of 0.9359 and 0.9765 respectively (Fig 4). The ordination patterns with the small sub-sample showed that samples with respect to bird age were separated on axis 2 while the A and H samples were separated from all other samples on axis 1 (Fig 4A). The ordination patterns with the large sub-sample revealed that samples were separated on axis 1 with respect to age; and the A samples were separated from all other samples on axis 2 (Fig 4B). Irrespective of the number of sequences sub-sampled, a clear spatial separation based on bird age, but not breeder source was observed.

The results of AMOVA (Table 2) revealed that (a) the A group was significantly different from all other groups except the H group, (b) the H group was significantly different from all groups except the A group, (c) the 2W, 4W and 6W groups from the monitored flock were significantly different from each other, (d) for the control flock, the 4W_C group was significantly different from 6W_C group, and (e) between the monitored and control flocks, only the 4W group was significantly different from 4W_C group. These results confirmed that the A and H groups were different from all the other groups as observed in NMDS plots (Fig 4A). These results also indicated that age, and not the breeder source, had a significant effect in spatially separating the groups on NMDS plots (Fig 4A and 4B).

**Table 2:** Results of AMOVA on the Bray-Curtis dissimilarity indices to determine the statistical significance of the spatial separation seen on NMDS plots.

	A	2W	4W	6W	2W_C	4W_C	6W_C	Sub-sampled sequences
**2W**	0.004							270349
**4W**	0.008	0.015						270349
**6W**	0.002	0.025	0.013					270349
**2W_C**	0.014	0.394	0.02	0.082				270349
**4W_C**	0.008	0.462	0.014	0.018	0.139			270349
**6W_C**	0.008	0.055	0.162	0.314	0.088	0.02		270349
**H**	0.077	0.002	0.005	0.003	0.001	0.014	0.024	137668

The *p* < 0.05 was considered significant.

### Differential abundance

**Fig 5:**
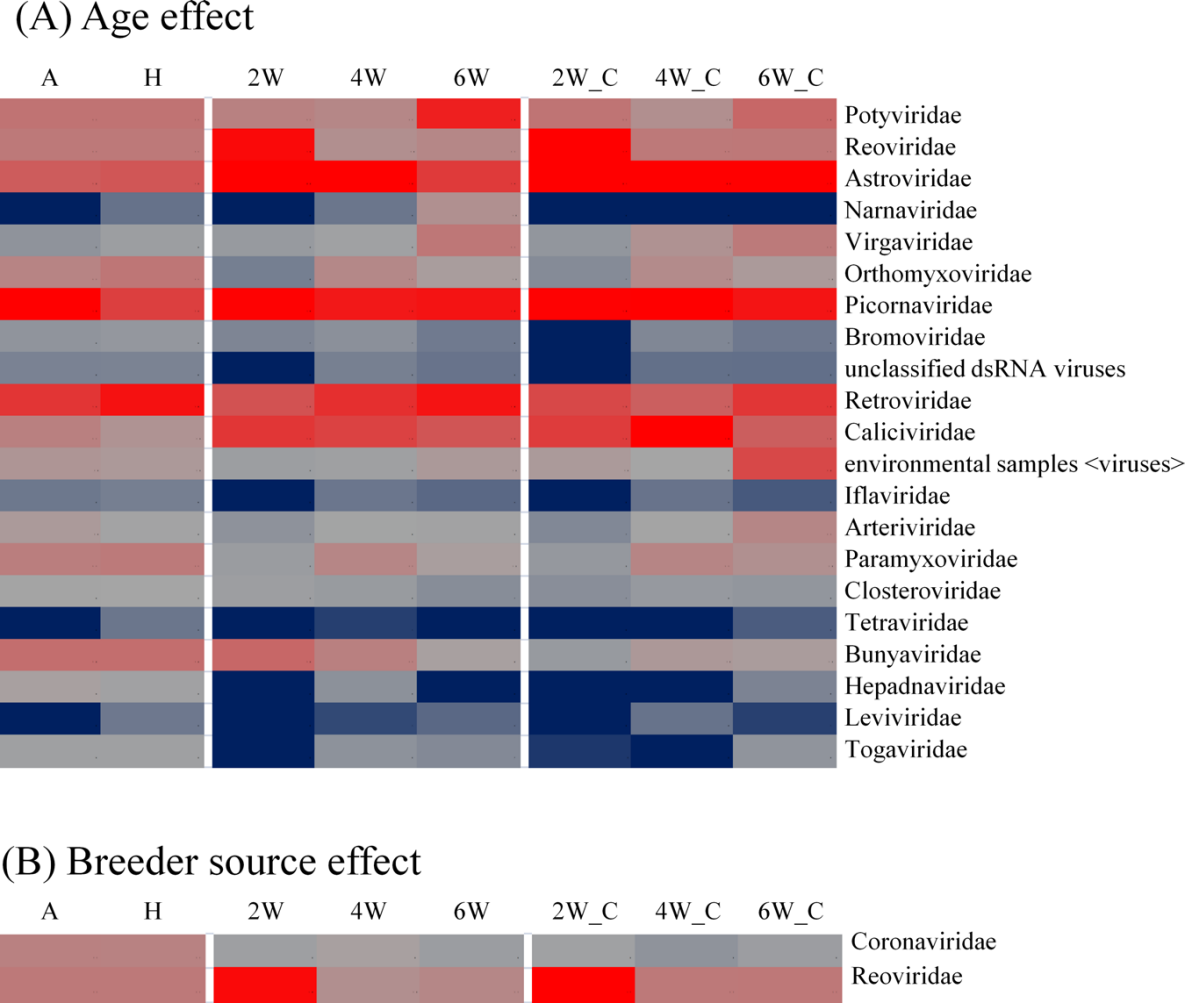
Differential abundance analysis. Differential abundance (*q* < 0.1) of RNA viral families in progeny birds from breeder A vs. breeder mix (which excluded A) over different ages (2, 4 and 6 weeks of age). The differential abundance was computed using the edgeR package. For each RNA viral family, the median values of all birds in each group are used for heat map representation. The abundance of RNA viral families in A and H samples is plotted only for comparison. The blue-gray-red color scale represents low to high abundance.

A total of 333 genera belonging to 80 RNA viral families were detected across all the samples. Differential abundance analysis at the family level showed 21 viral families based on bird age (Fig 5A) and two families based on breeder source (Fig 5B) were differentially abundant at FDR of 10% (*q* < 0.1). The RNA virus family Reoviridae was differentially abundant due to both age and the breeder source effects and was highly abundant at 2 weeks of age. The abundance of families such as Narnaviridae, Virgaviridae, Iflaviridae, Leviviridae, Togaviridae and unclassified RNA viruses, with very low abundance at 2 weeks of age, increased with age (Fig 5A). These results reaffirmed our AMOVA and NMDS results.

### Abundance profiles

**Fig 6:**
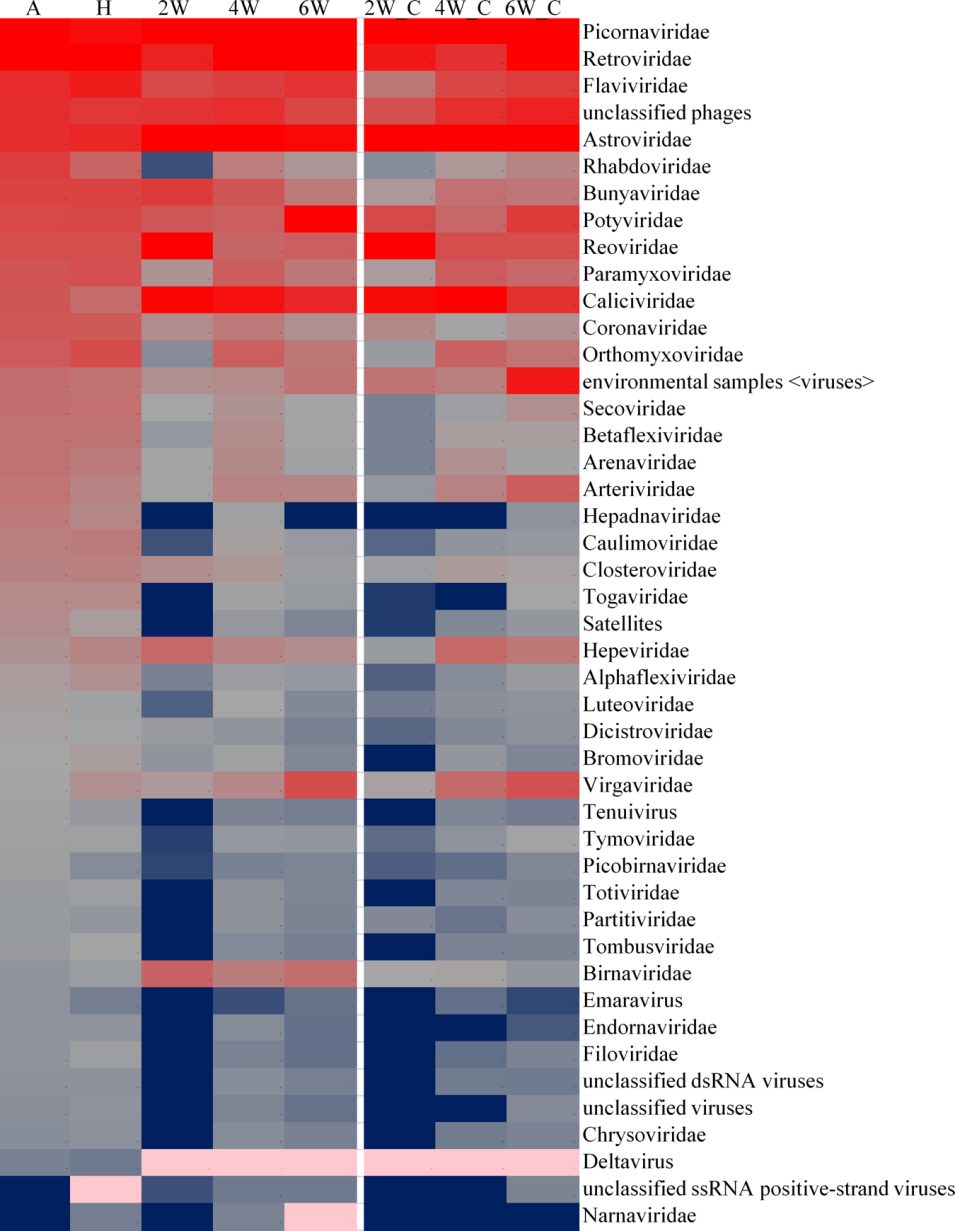
Distribution of abundant RNA viral families in all groups. For each RNA viral family, the median values of all birds in each group is used for heat map representation. The blue-gray-red color scale represents low to high abundance.

The median of the abundance values was first calculated for each viral family followed by a calculation of the median of all values in the family-group matrix. Of 80 viral families detected, 45 had at least one value equal to or greater than median of all the values in the family-group matrix, and these were considered to be abundant (Fig 6). The abundance distribution of the families in the A and H groups were similar to each other but very different than the birds from the test and control flocks at 2, 4 and 6 weeks of age. The abundance distribution of families in test and control birds at 2, 4 and 6 weeks of age were similar to each other. Many of the RNA viral families were present in low abundance in progeny birds at 2 weeks of age from either breeder source but their abundance increased with age. The top four highly abundant RNA viral families detected across all groups were Picornaviridae, Retroviridae, Flaviviridae and Astroviridae (Fig 6).

**Fig 7:**
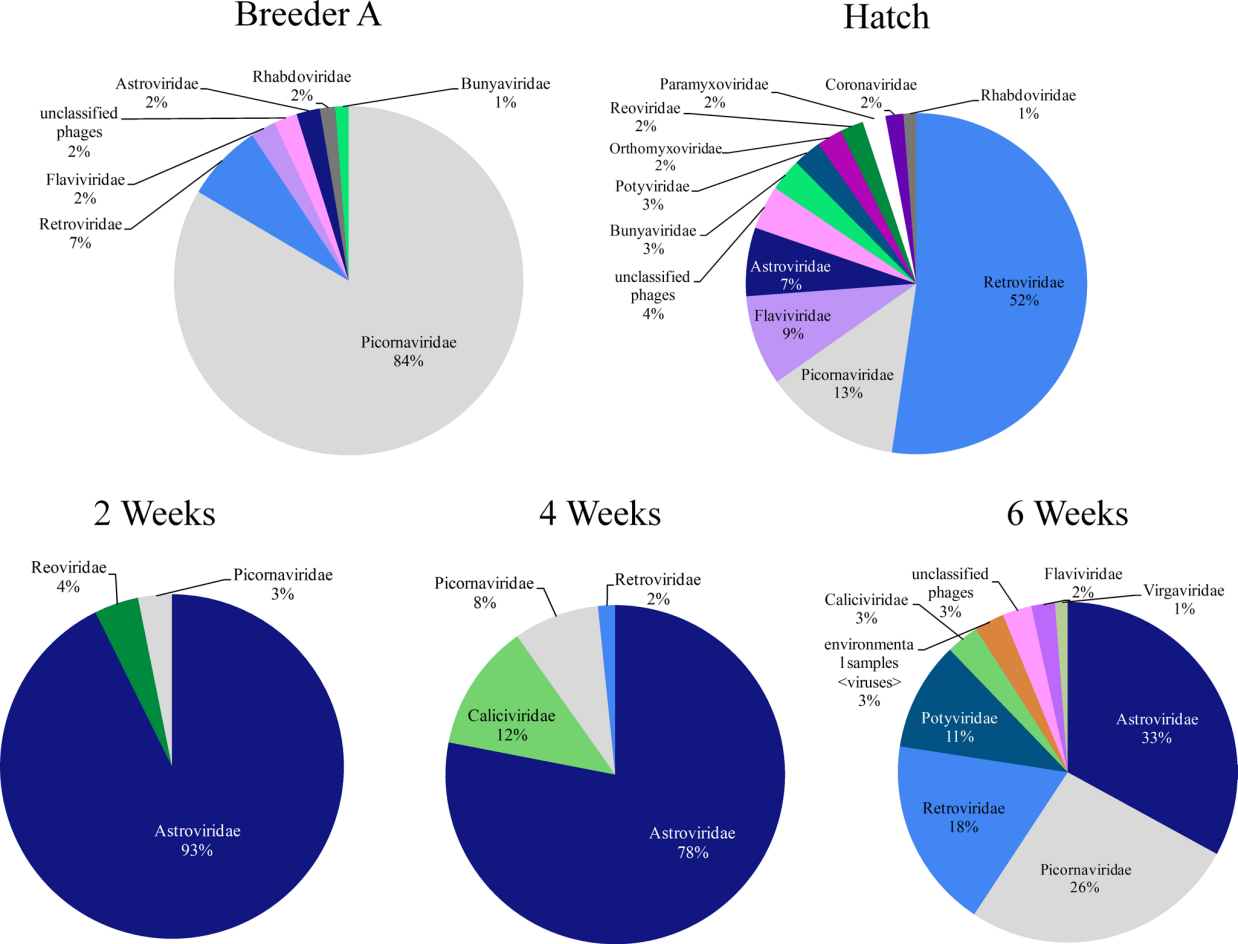
Abundance profiles for each age group. For each RNA viral family, the median values for all the birds in each age group, irrespective of the breeder source, were used to calculate the percentage and only RNA viral families > 1% abundance are used for pie chart representations.

The median abundance values for all birds were calculated for each virus family. Those which were present at greater than 1% in an age group are represented in Fig 7. The RNA viral families present in greatest abundance in the A and H groups were Picornaviridae and Retroviridae respectively; whereas the RNA viral family most abundant in 2, 4 and 6 week old birds was Astroviridae (Fig 7). Paramyxoviridae and Coronaviridae were also present in H group samples and their presence was detected most likely because of the vaccination for Newcastle disease and Infectious bronchitis viruses respectively in the hatchery [18]. The abundance of Astroviridae decreased with age while the abundance of Picornaviridae increased with age. The Retroviridae first appeared at 4 weeks of age and its abundance increased with age. Overall, total RNA viral diversity increased with age.

## Discussion

It is easy to understand how bacteria can be linked to animal health and are part of a normal healthy state. RNA viruses, on the other hand, infect the host, presumably to cause disease. The recent explosion of information on the microbial communities of animals has created a new perspective on what happens in healthy animals leaving open to question on the potential involvement of RNA viruses in health. Our findings demonstrate that not only are RNA viruses present in healthy animals but also that their community composition changes and diversity increases as birds age. The number of RNA viral families with > 1% abundance increased from three at 2 weeks of age to nine at 6 weeks of age, while the birds remained healthy. The Astroviridae, Picornaviridae and Retroviridae appeared to be the core members of the broiler gut RNA virome across all ages. The detection of Astroviridae family is in line with the findings of other investigators reporting the widespread prevalence of astroviruses in commercial chicken and turkey flocks from 2 to 6 weeks of age [12,13].

Enteritis is an age related disease in poultry, most often occurring between 2 and 4 weeks of age. In this study we found that at these ages, healthy broiler chicks already have an abundance of Astroviridae. So, although Astroviruses have been identified as etiologic agents of enteritis [8], our data suggest additional factors might be at play in development of clinical symptoms as other studies have suggested [12]. These findings may also suggest that the period of enteritis is coincidental with a period of low viral diversity in the gut which may be an indicator of host susceptibility. However, the temporal progression of RNA viral families in the poultry gut suggest that many of the paradigms of viral enteritis need to be reconsidered in light of this new information. Additional studies in which the progression of viral communities in flocks with viral enteritis and healthy flocks are compared are warranted.

The question of maternal influence on the bacterial community composition of progeny has been studied in mammals [19] but impacts on the wider microbial community, which includes viruses, has not been studied. Of course, mammals differ from birds in their relationship to their progeny and the link, for example, between bacterial community composition and type of birth [19], is not a factor in birds. In commercial settings like the one in this study, broiler eggs are removed from the breeder flock and taken to a hatchery where they are incubated and hatched and then to a third location where the progeny are raised. Given this process, it is likely that maternal influences on bacterial communities and also viral communities are different than in mammals. In this study, the RNA viral abundance profile, community distribution and composition of the samples from chicks on the day of hatch (H) was similar to that of their dams (A) but at 2, 4 and 6 weeks of age, it was more similar to their environmental neighbors i.e. the control flock. Based on these results we conclude that the breeder source influences the composition and structure of the RNA viral community at hatch but that the development of the RNA viral community after that is more strongly influenced by the bird’s environment. In addition, while the hatch samples were most similar to the breeder source, they also contained viral RNA from families Paramyxoviridae and Coronaviridae, which were not detected at any other age. We attribute the presence of these RNA viruses to vaccination at the hatchery.

Studies on RNA viromes in animal hosts are new and will require many iterations before a full picture of the biology of the interactions between viral families and their hosts will emerge. This study provides a baseline looks at RNA viral communities and their progression in the broiler chicken host.
